# THE PUBLIC HEALTH IMPLICATIONS OF DEMENTIA CAREGIVING: NATIONAL AND STATE-LEVEL VARIATIONS

**DOI:** 10.1093/geroni/igae098.1932

**Published:** 2024-12-31

**Authors:** Joseph Gaugler, Elma Johnson, Maya Koffski, Benjamin Denno, Matthew Baumgart, Basia Belza, Lisa McGuire

**Affiliations:** University of Minnesota, Minneapolis, Minnesota, United States; University of Minnesota, Minneapolis, Minnesota, United States; University of Minnesota School of Public Health, Minneapolis, Minnesota, United States; Alzheimer’s Association, Chicago, Illinois, United States; Alzheimer’s Association, Chicago, Illinois, United States; University of Washington, Seattle, Washington, United States; GSA, Atlanta, Georgia, United States

## Abstract

Unpaid dementia care has emerged as a public health priority. Specifically, dementia caregiving influences the health of those providing assistance (e.g., family members, friends) and recipients of care. However, the intensity of care provision and its health implications are often disproportionate within the population. Public health can engage in various initiatives to address such disparities, including improving surveillance of dementia caregivers with greater precision; engaging in outreach and education about the potential health risks of dementia care; and coordinating initiatives across traditional silos (e.g., public health, aging service providers, healthcare systems) to result in more available, accessible, and ultimately effective dementia care. Relying on 2021-2022 data from the Behavioral Risk Factor Surveillance System data from 47 states, we conducted a series of three-way cross-tabulations between dementia caregivers, non-dementia caregivers, and non-caregivers to ascertain key disparities and contextual factors that could serve as subsequent health targets. Nationally, although dementia caregivers provided more extensive help to care recipients for longer periods, variations in other domains (e.g., lifestyle behaviors) were less apparent nor consistent. More crucially, those patterns of statistical differences that emerged in the national analysis were far more variable and diverse when considering specific states, suggesting the urgent need for granular, more local data sources to better inform services, supports, and public health action to support dementia caregivers.

